# Planning sentence production in aphasia: evidence from structural priming and eye-tracking

**DOI:** 10.3389/flang.2023.1175579

**Published:** 2023-06-22

**Authors:** Willem S. van Boxtel, Briana N. Cox, Austin Keen, Jiyeon Lee

**Affiliations:** Aphasia Research Laboratory, Department of Speech, Language, and Hearing Sciences, Purdue University, West Lafayette, IN, United States

**Keywords:** aphasia, grammatical encoding, eye tracking, structural priming, sentence production, sentence planning

## Abstract

**Background:**

Grammatical encoding is impaired in many persons with aphasia (PWA), resulting in deficits in sentence production accuracies and underlying planning processes. However, relatively little is known on how these grammatical encoding deficits can be mediated in PWA. This study aimed to facilitate off-line (accuracy) and real-time (eye fixations) encoding of passive sentences through implicit structural priming, a tendency to better process a current sentence because of its grammatical similarity to a previously experienced (prime) sentence.

**Method:**

Sixteen PWA and Sixteen age-matched controls completed an eyetracking-while-speaking task, where they described a target transitive picture preceded by a comprehension prime involving either an active or passive form. We measured immediate and cumulative priming effects on proportions of passives produced for the target pictures and proportions of eye fixations made to the theme actor in the target scene before speech onset of the sentence production.

**Results and conclusion:**

Both PWA and controls produced cumulatively more passives as the experiment progressed despite an absence of immediate priming effects in PWA. Both groups also showed cumulative changes in the pre-speech eye fixations associated with passive productions, with this cumulative priming effect greater for the PWA group. These findings suggest that structural priming results in gradual adaptation of the grammatical encoding processes of PWA and that structural priming may be used as a treatment component for improving grammatical deficits in aphasia.

## 1. Introduction

Successful sentence production is associated with careful encoding of a message into a grammatical structure. Although various models of grammatical encoding exist, most models assume anticipatory stages of processing in which the to-be-produced sentence is, to some extent, planned out (see, Ferreira and Slevc, 2007; Thompson et al., 2015, for a review). Persons with aphasia (PWA) often experience problems with grammatical encoding resulting in impaired sentence production (see, Goodglass et al., 1979; Linebarger et al., 2000; Rochon et al., 2005, for examples). Broadly, theories on grammatical encoding in aphasia have focused either on a loss of syntactic knowledge—or parts thereof—as the underlying cause for encoding impairments (e.g., Grodzinsky, 2000; Friedmann, 2002), or on impairments in the use of or access to that syntactic knowledge (Schwartz et al., 1994; Marshall, 1995; Hartsuiker and Kolk, 1998). However, despite extensive research, the underlying causes of this impairment and how the grammatical encoding process could be facilitated in PWA are not fully understood.

The current study investigated anticipatory planning processes as a window into grammatical encoding in PWA, using an eye-tracking-while-speaking methodology and aiming to facilitate both off-line sentence production and real-time planning processes through a structural priming paradigm. Eye-tracking methodology has been applied extensively to study *comprehension* of grammar in PWA (e.g., Meyer et al., 2012; Hanne et al., 2015; Schumacher et al., 2015; Mack et al., 2016; Sharma et al., 2021), however studies of eye movements during sentence *production* in PWA are more scarce (cf. Cho and Thompson, 2010; Lee and Thompson, 2011; Lee et al., 2015; Lee, 2020). Nevertheless, analyses of eye movements have great potential to elucidate processing strategies in PWA, especially for more difficult sentence types (Griffin and Bock, 2000).

Structural priming, the tendency to repeat grammatical structures experienced before, is also associated with faster and less effortful processing of repeated structures and is known to support implicit language learning in healthy adults (see, Pickering and Ferreira, 2008, for a review). Promising early work suggests structural priming may be an effective method to facilitate production of sentences that are otherwise difficult to produce in PWA (e.g., Cho-Reyes et al., 2016; Lee and Man, 2017; Lee et al., 2019a). However, little is known on if, and how, structural priming can support real-time grammatical encoding processes in PWA (and healthy adults). If both sentence production and real-time planning processes, as measured by changes in eye fixations, can be modulated by structural priming in PWA, this would have important implications for the clinical translation of structural priming as a treatment in aphasia. It would further suggest that underlying representations of syntax are not lost in aphasia, but that their accessibility can be improved through training (Schwartz et al., 1994; Linebarger et al., 2000).

### 1.1. Grammatical encoding in aphasia

Before speakers begin production of speech, they plan their utterances in advance to at least some degree (e.g., Bock et al., 2003; Bock and Ferreira, 2014; Konopka and Meyer, 2014; Castellucci et al., 2022). The scope of these anticipatory encoding processes may be dependent on a speaker’s cognitive or linguistic capacity (Swets et al., 2014; Lee et al., 2015), ease of language formulation or processing load (Van de Velde and Meyer, 2014; Barthel and Sauppe, 2019), and conversational context (Swets et al., 2013), among other factors. Past studies have focused on analyses of pause rates and durations as indices of sentence planning (e.g., Lee et al., 2019b; Krivokapić et al., 2022), but innovative paradigms such as structural priming (e.g., Hardy et al., 2020) and eye-tracking-while-speaking (e.g., Lee et al., 2015) have also informed the scope and efficacy of sentence planning.

Monitoring speakers’ eye fixations to different actors in a to-be-described scene before speech onset can reveal how speakers use different grammatical encoding strategies (Griffin and Bock, 2000; Bock et al., 2003; Van de Velde and Meyer, 2014; Lee et al., 2015; Henderson, 2017). Speakers may plan their speech in a word-by-word manner, with little “lookahead” of other elements in the event. In such cases, speakers might show preferential looks to one element in a visual scene from the picture onset and continue to produce that element as the subject of the sentence (Griffin, 2001; Gleitman et al., 2007). In structure-driven planning, however, speakers show advanced planning of multiple message elements before speech onset. For example, when describing a transitive event, speakers may show non-preferential fixations to both agent and theme to “appraise” a causal relationship, before deciding on a “suitable” subject for the sentence (Griffin and Bock, 2000; Bock et al., 2003; Van de Velde and Meyer, 2014).

Less is known about real-time grammatical encoding processes in PWA. Albeit few, published studies suggest that advanced planning of message elements might be important for successful sentence production in PWA, especially when sentences involve complex grammatical encoding. For example, Lee et al. (2015) found that PWA incrementally planned utterances word-by-word, much like healthy controls, when producing lexical items in a predetermined word order (e.g., The clock and the bed are above the needle). However, when production tasks involved generation of sentence structures, PWA showed advanced planning of verb predicate information before speech (see also Lee and Thompson, 2011). In Mack et al. (2017), a group of PWA received a 12-week program of Treatment of Underlying Forms (TUF) to improve production of passive sentences. At pre-treatment, PWA showed abnormal eye fixations and very few productions of passive sentences. However, at post-treatment, improved production of passive sentences in PWA was associated with eye fixations reflecting structural planning. Their PWA showed equal fixations to both actors before they encoded the theme as the subject of the passive sentence. Similarly, Lee (2020), in examining how PWA and controls flexibly produce either active or passive sentences in response to lexical (agent, theme) priming, found that PWA, in their early anticipatory fixations, spent more equal looking time to both elements, while controls showed word-by-word driven planning. Early, pre-onset fixations are, in short, key to understanding grammatical encoding processes in PWA and there is evidence that real-time encoding processes can be trained in PWA after extensive therapy. This study focuses on if and how implicit structural priming experiences induce changes in real-time grammatical encoding processes in PWA within a single experimental session. If structural priming creates measurable changes in the real-time grammatical encoding processes in PWA, this could have important clinical implications.

### 1.2. Structural priming

Structural priming occurs when processing of a grammatical structure facilitates subsequent processing of the same structure and biases language users to the use of that structure (Bock, 1986; Pickering and Ferreira, 2008). Structural priming is a robust effect in production, where speakers show greater tendencies to produce primed structures (e.g., Bock, 1986; Griffin and Weinstein-Tull, 2003; Kaschak et al., 2011, among many others). For example, after reading or hearing a passive sentence prime, speakers are more likely to produce a passive rather than an active structure to describe a new transitive event. Effects are amplified when lexical information is shared between primes and targets, an effect known as the lexical boost (Hartsuiker et al., 2008; Traxler et al., 2014). In comprehension, facilitation effects by structural priming can be observed through more efficient eye fixations while listening or reading (e.g., Thothathiri and Snedeker, 2008; Traxler et al., 2014), reading speeds on self-paced reading measures (Van Boxtel and Lawyer, 2022), or decisions on sentence-picture matching or attachment ambiguity tasks (Pickering et al., 2013).

Structural priming effects are, however, not mere repetition, but reflective of experience-based tuning in the central syntactic system. This is evidenced by cross-modal structural priming, from comprehension to production and vice versa. For example, Bock et al. (2007) presented auditory prime sentences to participants, following which they described a presented picture. Persistent evidence of structural priming was found even when participants did not repeat the prime sentences out loud, demonstrating that comprehension of a prime influences subsequent production (for similar results, see Branigan et al., 2000; Pickering and Garrod, 2004; Branigan, 2007; Segaert et al., 2012; Tooley and Bock, 2014). Recent findings show cross-modality priming can be effective in PWA as well.

For instance, Man et al. (2019) took turns with PWA to describe pictures in a dialogue-like game, and found a clear structural priming effect in both PWA and healthy controls. Keen and Lee (2022) report findings of production-to-comprehension priming in PWA in an ambiguous clause attachment paradigm. Both PWA and healthy controls were found more likely to interpret ambiguous attachment sentences using the attachment preference following a priming phase where they produced sentences with this specified preference. This indicates that syntactic representations are shared between production and comprehension, and that these representations are not lost or damaged in aphasia.

Importantly, structural priming has also been reported as a gradual facilitatory effect across one or several experimental sessions. Such cumulative priming is crucial to understanding how priming leads to lasting, persistent changes to the language production and/or comprehension systems. Short-term, immediate priming effects can be captured by the likelihood of a speaker describing, for instance, a transitive scene with a passive following a passive prime. On the other hand, cumulative effects would include the cumulative proportion of passives out of all transitive responses across an experimental session (see Heyselaar et al., 2021). Cumulative measures are therefore crucial in determining whether the syntactic system shows gradual adaptation through structural priming. In addition to a gradual increase in target structure productions, cumulative effects of structural priming have also been reported on eye movements. For instance, in a reading study with healthy adults, Tooley and Traxler (2018) found shorter eye fixation times on ambiguity–resolving regions in reduced relative sentences (e.g., “The dog *kicked by* the boy was sad”) as priming progressed across five sessions, indicating that the syntactic system was being “trained” to process this complex structure more effectively. Cumulative priming in aphasia is nevertheless poorly understood. Early research by Saffran and Martin (1997) reported PWA produced more target dative structures following priming training than beforehand, an effect which was replicated more recently by Lee and Man (2017). However, to our knowledge, no study has reported online, eye fixation measures to evaluate syntactic priming in aphasia.

Another question that deserves further investigation involves lexical boost effects on structural priming. When prime and target share lexical material, e.g., a verb, studies with healthy speakers have shown greatly increased magnitudes of priming than without such overlap (Hartsuiker et al., 2008; Van Boxtel and Lawyer, 2022). This suggests representations of syntactic structure may be linked to, or reinforced by, lexical information. However, only three studies thus far examined lexical boost in PWA and yielded conflicting findings. These studies used distinct priming methodologies. Yan et al. (2018) found intact lexical boost effects on production of transitive sentences in PWA. Their priming task obligated participants to repeat the prime sentence and then compare their own repetition with the written prime sentence prior to target picture description. However, Man et al. (2019) failed to find lexical boost effects on the production of transitive and dative sentences in PWA in a dialogue-like priming task, where their PWA simply heard their interlocutor (experimenter’s) picture descriptions as primes prior to their turn to describe target pictures. Similarly, Lee et al. (2019a) did not find lexical boost effects in either the control or PWA group using a comprehension-to-comprehension priming task involving written sentences with ambiguous prepositional phrase (e.g., “the cop is poking the waitress with an umbrella”). Evidence on whether, and under what circumstances, the verb overlap amplifies structural priming in aphasia is therefore very limited and requires more research. The current study used a priming methodology which has not yet been used in previous studies, an auditory sentence-to-picture matching task.

### 1.3. The present study

This study aimed to facilitate grammatical encoding in PWA and age-matched controls through structural priming, focusing on off-line (picture description) and on-line (eye fixation) measures. We presented PWA and controls with auditory active or passive sentence-picture matching primes, following which they described a transitive picture while eye movements were recorded. Three questions were investigated. First, we asked whether PWA and controls would show immediate priming effects on their off-line production and eye fixations. We expected PWA and controls to show a greater likelihood of producing passives and looking at the theme actor after comprehending a passive compared to an active prime, although the effects might be smaller in PWA, following earlier studies (Yan et al., 2018; Man et al., 2019). In addition, we examined if verb overlap between prime and target enhanced priming effects. Given the contention around lexical boost effects in PWA, we made no specific predictions about whether our patient group would show lexical effects. Lastly and most importantly, we sought novel evidence for whether structural priming creates cumulative adaptation in the grammatical encoding processes of PWA and controls. In line with implicit learning views of structural priming (e.g., Bock and Griffin, 2000; Chang et al., 2006), we hypothesized that PWA and controls would show increased production of passive sentences as the experiment progresses and cumulative changes in the pre-speech eye fixations associated with passive productions.

## 2. Materials and methods

Eighteen persons with stroke-induced aphasia (PWA) and nineteen age-matched healthy controls took part in the study and were compensated for their time. Due to excessive artifact on eye movement recordings, 3 control participants’ data was removed from the study; two PWA were further eliminated from the study due to below chance level scores on comprehension of prime sentences (33 and 23%, respectively). All participants were native monolingual speakers of English with normal or corrected-to-normal vision and hearing, and all passed hearing screenings. Participants provided informed consent before taking part in the study, which received approval from the local institutional review board. Patient group data were stored and accessed in line with the U.S. Health Insurance Portability and Accountability Act and all participants’ data were kept securely on password-protected and encrypted drives and in locked storage cabinets accessible only to members of the research team.

Control participants ranged from 50 to 84 years of age (*M* = 60.8; SD = 8.99; [50,84]) and all scored within normal limits on the Cognitive Linguistic Quick Test (Helm-Estabrooks, 2001), measured by composite severity rating (*M* = 3.95; SD = 0.25; [3.6,4]). The control group was generally well-educated, with an average 17.25 years spent in formal education (SD = 1.69; [12,20]). The control and PWA groups did not differ in terms of age [*M*_Control_ = 60.8, SD = 9.0; *M*_PWA_ = 64.9, SD = 12.7; *t*_(27)_ = −1.06, *p* = 0.299], though the control group spent more years in education [*M*_Control_ = 17.3, SD = 1.7; *M*_PWA_ = 14.5, SD = 2.1; *t*_(29)_ = 4.08, *p* < 0.01].

Participants with aphasia required a diagnosis of aphasia secondary to a left-hemisphere stroke at least 6 months prior to the study to be eligible for participation. Aphasia profiles of PWA in this study included a mix of non-fluent Broca’s aphasia and some fluent (Wernicke’s, anomic) aphasia. On average, PWA in this study were 60.3 months post stroke onset (SD = 40.1), and none reported a history of speech or language impairments prior to stroke. PWA completed the Western Aphasia Battery (WAB; Kertesz, 2006) and the Northwestern Assessment of Verbs and Sentences (NAVS; Thompson, 2012). Scores on these tests and their sub-tests are given in Table 1. We included PWA whose auditory comprehension composite score was higher than 7/10 on the WAB-R to ensure that their comprehension of sentence-level stimuli was relatively preserved. On the NAVS, all PWA showed higher than chance level performance on the comprehension of single verbs (VCT) and sentences with canonical word order (SCT-C). Their performance on the production tests, including Verb Naming, Argument Structure Production, and Sentence Priming Production, varied across individuals. However, all participants demonstrated ability to produce some verbs and simple sentence-level (intransitive, transitive active) utterances.

### 2.1. Materials

#### 2.1.1. Prime and target sentences

This study used a comprehension–to–production priming paradigm, where listening to active or passive prime sentences in a sentence-picture matching task was followed by participant’s descriptions of pictures depicting transitive events. To this end, we used twelve transitive verbs (e.g., pull, chase) in conjunction with 39 animate nouns (e.g., dog, chef) to create a total of 48 prime–target pairs. Nouns were used a maximum of 12 times across all stimuli (*M* = 4.79, SD = 3.10) and were equally distributed across prime conditions. Nouns were further balanced within the stimuli such that no nouns were disproportionately used in either an agent or theme position (*M*_Frequency as Agent_ = 1.23, SD = 1.37; *M*_Frequency as Theme_ = 1.15, SD = 1.09; *t* = 0.782, *p* > 0.05) All verbs were kept to one or two syllables in length and had a mean log-lemma frequency of 1.536 (SD = 0.56; Baayen et al., 1996). A full list of experimental sentences used can be found at https://osf.io/7apc3/.

**FIGURE 1:**
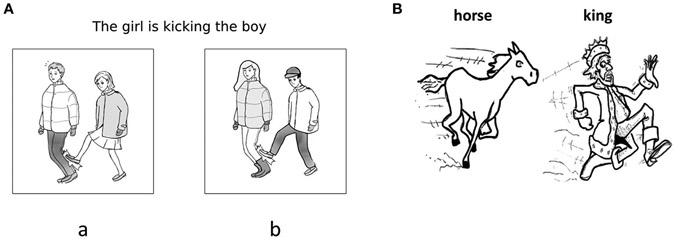
**(A, B)** Examples of target and prime displays. Prime screens were accompanied by auditory prompts matching either picture a or b, while target screens were preceded by a prompt reminding participants it was their turn to speak.

A single experimental trial consisted of a sentence-picture matching prime and a picture description target. Target items were paired with black-and-white line drawings corresponding to the action depicted in the sentence. To accommodate possible word retrieval impairments in PWA, written names of the agent and theme actors were provided on each target picture, as exemplified in Figure 1A. Primes were presented as a sentence–picture matching task paired with two pictures, one depicting the correct thematic roles of the characters, the other showing a role–reversed event. Figure 1B gives an example of this set-up. All pictures were counterbalanced for actor order, such that the agent appeared on the left side on half of all pictures and on the right side on the other half. We normed the target pictures used with a group of college-aged healthy speakers (*n* = 10) and found that the pictures elicited the correct actors and verbs 97% of the time.

Two filler items were included between each experimental trial (i.e., following each experimental prime-target sequence), for a total of 96 filler sentences. These fillers were presented in the same manner as prime items, as a sentence-picture matching task. Filler sentences comprised predicate structures (e.g., “There is a circle”) or intransitives (e.g., “The dog howls”).

#### 2.1.2. Design

Priming conditions were manipulated through auditory recordings paired with prime pictures. The example in Figure 1A could be paired with a passive prime sentence, “The boy is kicked by the girl”, or an active prime sentence, “The girl is kicking the boy”. It was hypothesized that participants should be more likely to describe target pictures with passive sentences after hearing a passive comprehension prime compared to an active comprehension prime. Half of all prime–target pairs were verb-matched, while the other half had different verbs to test lexical boost effects. If verb repetition boosts priming effects, participants should show increased priming effects following same-verb vs. different-verb comprehension Primes.

Thus, a 2x2 design was created with four prime conditions: (1) active, same-verb prime; (2) passive, same-verb prime; (3) active, different-verb prime; (4) passive, different-verb prime. For instance, for the target “horse—chase—king” in Figure 1B, auditory primes included sentences such as those in (1–4) below. Twelve trials were assigned to each condition, for a total of 48 trials. The experiment was subdivided into four blocks and the order of trials within blocks was pseudo-randomized so that no two trials in the same prime condition were presented consecutively. The presentation order of these blocks was counterbalanced across participants to avoid any potential of order effects.

1. The lion is chasing the woman (active, same–verb);
2. The woman is chased by the lion (passive, same–verb);
3. The lion is kissing the woman (active, different–verb);
4. The woman is kissed by the lion (passive, different–verb).

### 2.2. Procedure

Prior to the start of the study, participants were familiarized with the nouns and verbs used in the experiment using an oral reading task. Words were presented above corresponding line drawings and participants were asked to read the words aloud. In the case of errors, feedback was provided. This familiarization task was completed to ensure that the participants could read the individual words necessary for producing target sentences and minimize the potential influence of their word-finding difficulties.

**FIGURE 2:**
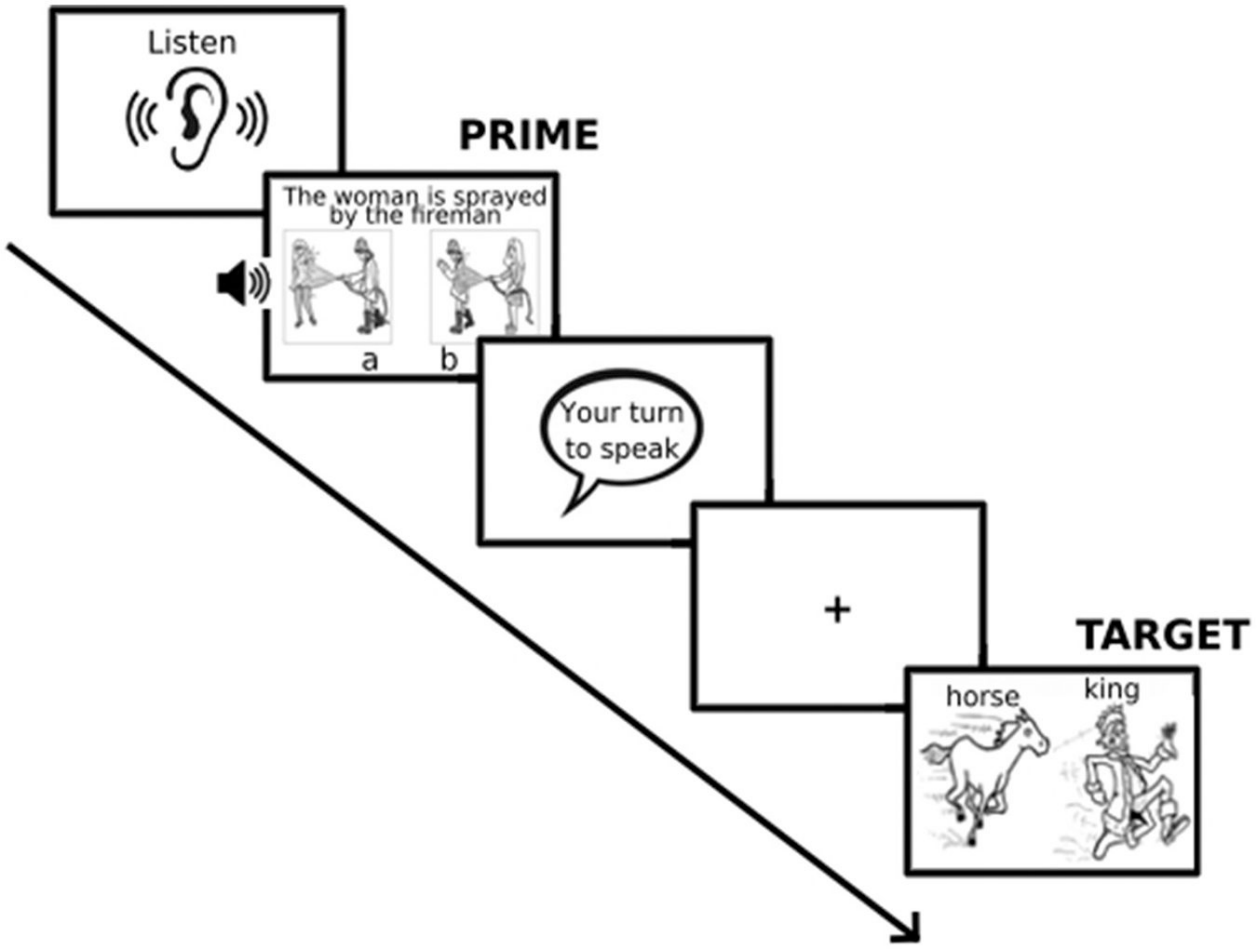
Sequence of experimental trials. The trial shown consists of a passive, different–verb prime.

The priming task began with instructions and 4 practice trials. As shown in Figure 2, participants were instructed to listen carefully to spoken sentences whenever a “Listen” symbol appeared on the screen, and to press the key corresponding to the picture which matched the spoken sentence. These keys were set as the Numpad 1 and Numpad 3 keys, which were physically overlaid with stickers reading “a” and “b”. Participants were further told to describe the picture on the screen after seeing a “Your turn to speak” speech bubble. A pure tone marked the onset of the Target picture presentation.

Participants proceeded through the experiment at their own pace by pressing the Space bar to advance from screen to screen. All stimuli were presented on a 23-inch BenQ computer screen with a refresh rate of 60 Hz, running Windows 10 Pro. Stimuli were prepared in Experiment Builder (SR Research Ltd, 2011, Missisauga, Ontario) and presented in black Arial font on a white background. Participants were offered the opportunity for a break between each experimental block; the full experiment took most participants between 40 and 60 min.

### 2.3. Recording and analysis

#### 2.3.1. Behavioral and speech time responses

Responses on prime sentences were scored as correct if participants selected the correct picture which matched the prime picture they heard. Only trials where participants correctly comprehended prime sentences were included in subsequent analyses (*M*_Control correct_ = 98.3%; *M*_PWA correct_ = 88.1%). For target productions, participants’ verbal responses were recorded using a Shure SM58 microphone connected to a PreSonus TubePreV2 preamplifier, and manually transcribed and coded for sentence structure (Active, Passive, or Other). Responses were scored as active if participants used an agent–verb–theme order, and as passive if a theme–verb–agent order was used. Substitutions of words with synonyms were allowed and did not affect accuracy scoring (e.g., “guy” for “boy”). Passive responses were scored correct regardless of the tense of the auxiliary verb (e.g., “the girl was/is chased by the boy”). However, correct verbal morphology (“–ed”) and a subsequent “by” prepositional phrase were required for scoring passive responses as correct. For actives, variations in verb tense inflections were accepted in both participant groups (e.g., *“punched, punches, is punching”*). In the case of PWA, omission of an auxiliary verb (e.g., “The king chased by the horse” and “The horse chasing the king”) and production of intelligible phonological paraphasias (e.g., “tasing” for “chasing”) were accepted. Where responses were self–corrected or multiple attempts were made, the first sentential response (consisting of at least a subject noun and a verb) was scored. All trials where neither an active or a passive structure was produced were removed prior to analysis (*M* = 13.5%). PWA showed higher overall production error rates than controls [*M*_Control Correct_ = 95.7%; *M*_PWA Correct_ = 77.3%; *t*_(16)_ = −3.51, *p* < 0.01].

In addition, speech onset times (SOT) were measured to align eye fixation data with regions of speech. Onsets of the first noun, the main verb, and the second noun in participants’ target responses were marked using Praat (Boersma and Weenink, 2022). These onset times were then used to align eye movement data (see below). For each participant and each trial, SOTs were measured by manual marking of word onsets. The full measurement protocol and an exploratory analysis of onset measurement in Praat can be found in our Supplementary material at https://osf.io/7apc3/. Overall, PWA showed slower onset times of the first noun compared to controls [*t*_(32)_ = 4.73, *p* < 0.001], as well as of the main verb [*t*_(33)_ = 5.84, *p* < 0.001] and the second noun [*t*_(32)_ = 5.66, *p* < 0.001]. However, no effects of syntactic or verbal overlap were evident on the SOT measures (all *p*s > 0.05).

#### 2.3.2. Eye movements

During target picture descriptions, eye movements were recorded with an EyeLink 1000 infrared tracker (SR Research Ltd), sampling at 1,000 Hz. Participants were seated in a room with constant lighting conditions, around 60 cm from the display monitor, while monocular eye movements were recorded, and were instructed not to move their heads or bodies during the study. A nine-point calibration was conducted before the experiment which was additionally validated at the beginning of each experimental block. A maximum measurement error of 1◦ for each calibration point was accepted. Eye movement recordings were visually checked for intactness in EyeLink Data Viewer 4.2.1 (SR Research Ltd, 2018), before fixations were calculated. Fixations were computed using the EyeLink standard algorithm, which employs a velocity and acceleration-based detection method. The resulting variables included fixation location (to either the agent or theme actors) and onset and offset times (allowing for the computation of fixation duration). As mentioned above, eye data were aligned to speech production through analysis of Speech Onset Times. This allowed for the analysis of eye fixations which happened before speech onset of the first noun.

#### 2.3.3. Statistical analysis

Statistical analysis was conducted in R 4.2.1 (R Core Team, 2022). and Rstudio 2022.07.1 (RStudio Team, 2020) running on a 64-bit Linux Ubuntu system. (Generalized) linear mixed models were computed in the *lme4* package (Bates et al., 2015) and evaluated using *lmerTest* (Kuznetsova et al., 2017). Model fit statistics were calculated with the *EMAtools* (Kleiman, 2021) and *MuMIn* (Barton, 2022) packages. Finally, figures were generated using *ggplot2* (Wickham, 2016), and confirmatory Bayesian models were computed using the *brms* (Bürkner, 2021) and *bridgesampling* (Gronau et al., 2020) libraries.

Fixations with a duration more than 2 standard deviations from each participant’s mean fixation duration were trimmed in order to improve the normal distribution of the data. Our analysis of fixations centered around the pre–onset region, which was defined according to the SOT data. This region included fixations with an end time before the production onset of the first noun. Previous eyetracking studies with both healthy speakers and PWA have shown that grammatical encoding of the sentential subject for active and passive alternations primarily occurs during the pre-onset region (Griffin and Bock, 2000; Bock and Ferreira, 2014; Lee, 2020; Weirick and Lee, 2022). For each trial, we computed the proportion of theme fixation time by dividing the total time spent fixating on the theme by the total time spent fixating either the theme or the agent.

We examined immediate priming effects on target picture descriptions as well as cumulative effects across the experimental session. Immediate priming effects were measured as the likelihood of passive picture descriptions following a passive comprehension prime, and increased proportions of looks to the theme. Predictor variables for immediate priming effects were dummy coded and all variable levels were compared against one another: Group (PWA vs. Control), Prime (Active vs. Passive), and Verb (Same vs. Different). Where possible, random effects were included in our models for Participant and Trial, and a covariate for Years in Education was also fitted wherever model fit allowed.

Cumulative effects were examined as follows. For behavioral responses, a *Cumulative Passive Proportion* variable was calculated as the proportion of passive target responses out of total active and passive responses produced up until the current target trial. For instance, if Participant A produced 10 active and 20 passive responses up until trial 30, their Cumulative Passive Proportion would be 20 / (10+20) × 100 = 66.67%. This approach mirrors that of Heyselaar et al. (2017), who used this cumulative variable to examine learning trends in groups of amnesic and control patients. To examine cumulative changes on fixation times, we calculated the proportion of fixation time spent looking at the theme actor in each trial. This proportion was then by target structure produced across the sequence of experimental trials. In both behavioral and eye-tracking cumulative models, trial order was included as a fixed effect, while prime condition was not.

Additional Bayesian models were computed to confirm the presence or absence of group effects. Supplementary analyses based on Bayes’ Factors (BFs) can express the likelihood of null findings with greater confidence than frequentist analyses (Wagenmakers et al., 2016). We computed models including interactions of group by prime and/or verb (depending on the manipulation of interest) as well as models including only simple effects of those parameters. Comparing these models yielded Bayes’ Factors, with values of >3 favoring the full model, including the interaction term, and smaller values of <1 favoring the null hypothesis, i.e., an absence of group effects (see, Lee and Wagenmakers, 2014, for a discussion). The code used for our supplementary Bayesian analysis can be found at https://osf.io/7apc3/.

## 3. Results

### 3.1. Immediate priming effects

#### 3.1.1. Behavioral responses

Generalized linear mixed models were fitted to predict active or passive responses on target productions, including a random effect for participant (adding another random effect for trial resulted in model convergence issues)—Table 2 shows a full summary of fixed effects. Generally, more passive targets were produced following passive primes (*z* = 5.405, *p* < 0.001, 95% CI [0.012; 0.044]), indicating a general priming effect. Controls produced more passives than PWA (*z* = 2.603, *p* = 0.009, 95% CI [0.003; 0.025]) and controls also produced more passives following passive primes than PWA (*z* = −3.634, *p* < 0.001, 95% CI [0.002; 0.021]). Pairwise Tukey-adjusted *post-hoc* analyses showed immediate priming effects were not significant in PWA alone (*t* = −2.02, *p* > 0.05). This was confirmed by our Bayesian models, which were highly suggestive of group differences on priming measures (BF > 1,000).

**FIGURE 3:**
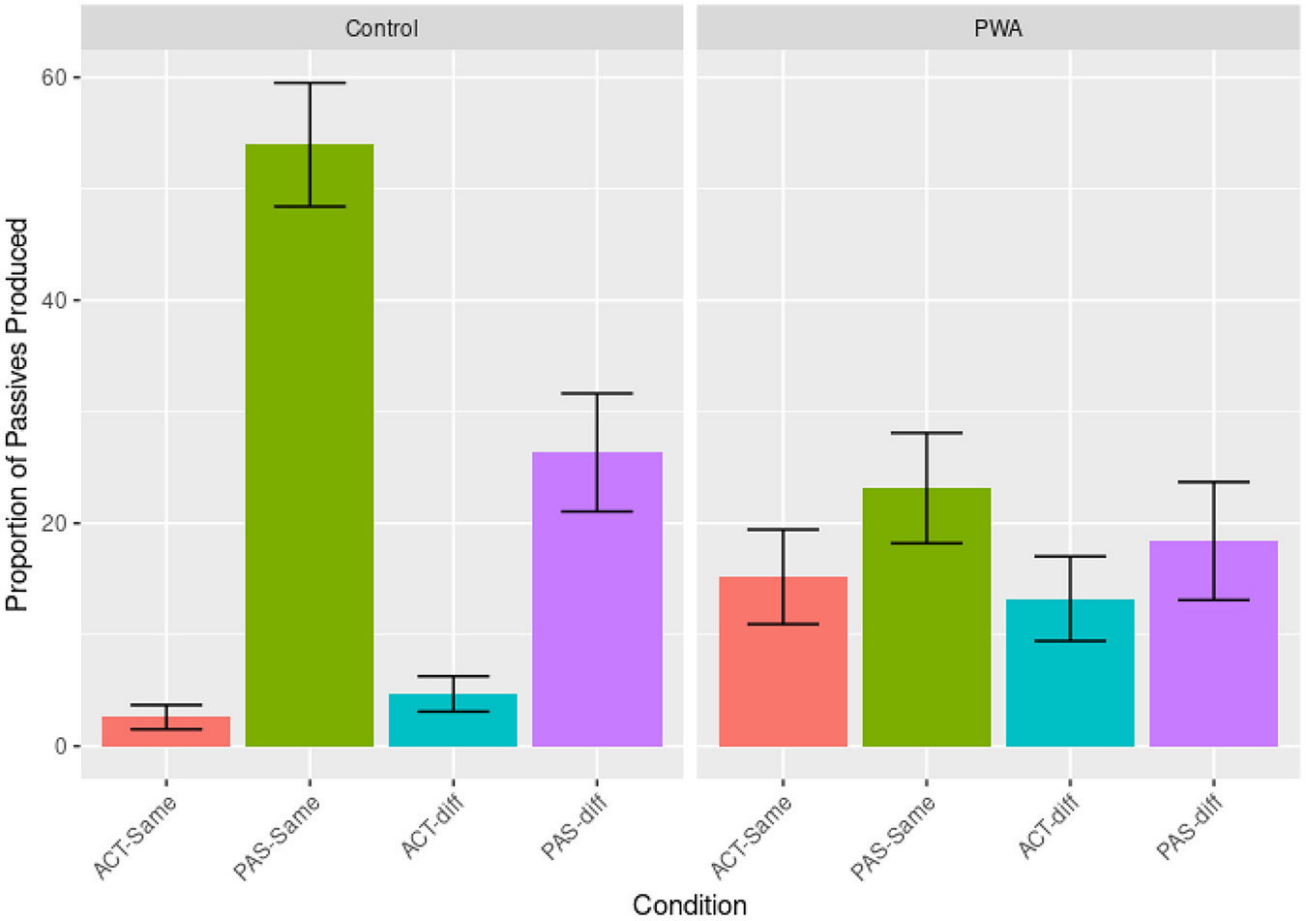
Plot of the proportion of passive targets produced by priming condition [ACT or PAS denoting active or passive prime, and same and diff(erent) denoting verb overlap] and group. Error bars represent average standard error of each participant’s mean.

Lexical overlap with primes increased the probability of producing a Passive target overall (*z* = 3.395, *p* = 0.001, 95% CI [0.004; 0.027]). *Post-hoc* analyses showed a higher probability of producing a passive following a same-verb passive prime compared to a different-verb passive prime (*z* = −4.544, *p* < 0.001). Different-verb passives were more likely to elicit a passive response than same-verb actives (*z* = −5.324, *p* < 0.001), but same-verb actives did not elicit more passives than different-verb actives (*z* = 0.892, *p* > 0.05). However, a group * prime * verb interaction showed lexical boost effects were driven by the control group (*z* = −2.620, *p* = 0.01, 95% CI [0.000, 0.015]). *Post-hoc* analyses confirmed lexical boost effects were not present in our PWA group (*z* = −0.822, *p* > 0.05), and Bayesian model comparisons further suggested group differences on lexical boost measures (BF_Verb*group_ = 19). Figure 3 illustrates priming effects.

#### 3.1.2. Eye fixations

**FIGURE 4:**
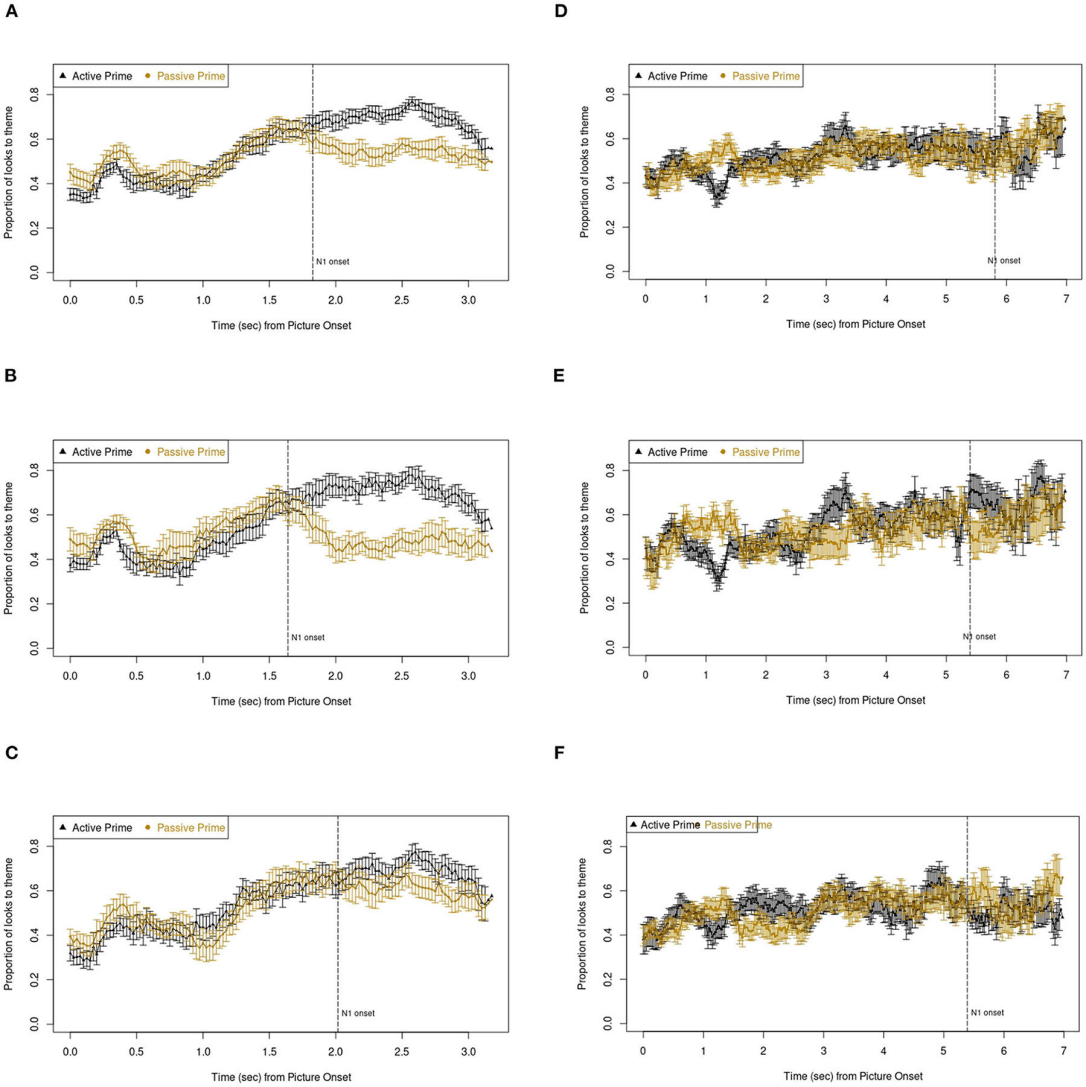
Plots showing the proportion of fixations to the theme by prime condition and verb condition relative to onset of the first noun. Error bars represent standard error of the mean. **(A)** Control: fixation to theme by prime condition. **(B)** Control: fixation to theme by prime condition in SAME-verb trials. **(C)** Control: fixation to theme by prime condition in DIFFERENT-verb trials. **(D)** PWA: fixation to theme by prime condition. **(E)** PWA: fixation to theme by prime condition in SAME-verb trials. **(F)** PWA: fixation to theme by prime condition in DIFFERENT-verb trials.

Linear mixed-effects models were fitted to eye fixation data in the pre-onset region (see Section 2.3). Specifically, the proportion of time spent fixating the theme in each trial was used as our dependent measure. These models therefore predicted the *relative* amount of time spent fixating the theme under different prime conditions. Fixed effects comprised a three-way interaction of prime by verb by group, and random effects of trial, participant, and agent position (whether the agent featured on the left or the right side of the target picture) were also included. See Table 3 for a full summary of this model. Figure 4 shows the time course of fixations to the theme by prime condition in either group.

A significant priming and lexical boost effect was observable, such that participants fixated the theme less when primed with a passive with a matching verb than with a different verb *t* = −2.032, *p* < 0.05, *d* = 0.073). The lexical boost varied by group, with weaker effects in the PWA compared to the control group (prime*verb*group: *t* = 3.009, *p* = 0.003, *d* = −0.040). Pairwise *post-hoc* comparisons suggested PWA showed a significant priming effect (*t* = −3.482, *p* = 0.003) but no lexical boost (*t* = 0.167, *p* > 0.05).

### 3.2. Cumulative priming

#### 3.2.1. Cumulative passive proportion

**FIGURE 5:**
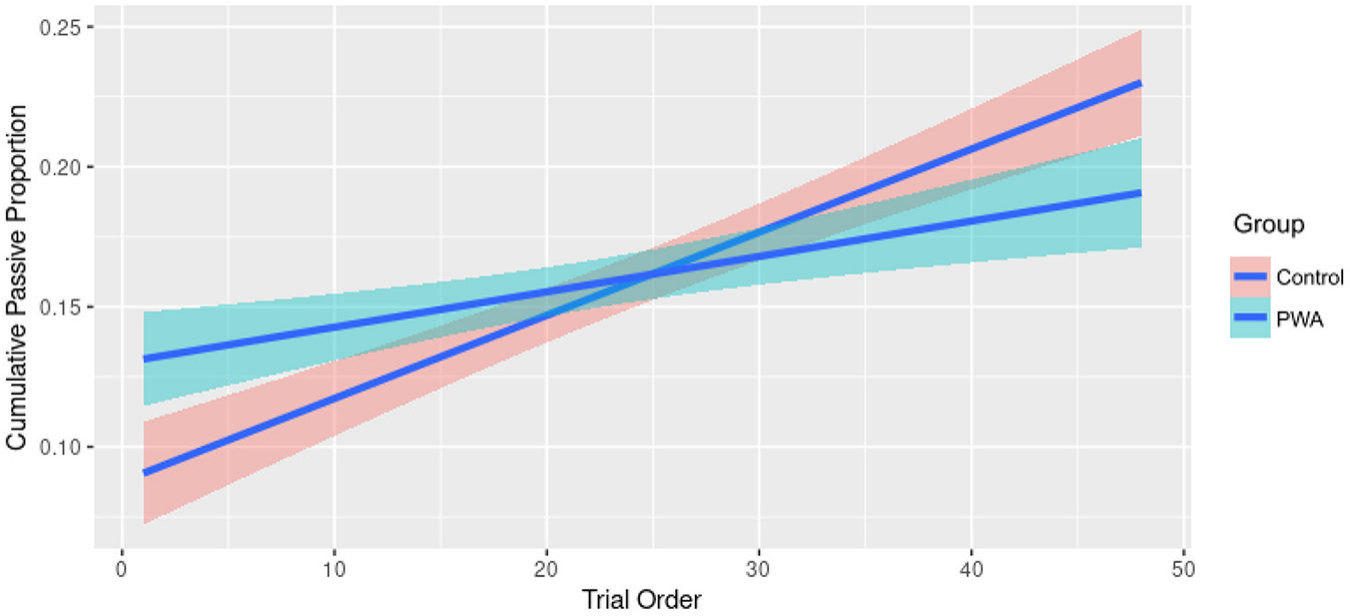
Plot showing the cumulative proportion of passives produced across experimental trials by participant group. Both groups produced more passives as the experiment progressed.

We also included Trial Order into models to investigate whether participants produce more passive sentences as the session progresses; i.e., cumulative priming effects. For the dependent measure, we computed the cumulative proportion of passives produced by participants across the experimental sequence. These data are visualized in Figure 5 and model outputs are given in Table 4. Crucially, across groups, participants produced more passives as the experiment progressed, indicating a strong cumulative priming effect (*t* = 15.76, *p* < 0.001. *d* = 0.842, 95% CI [0.003; 0.003]). This effect was stronger in the Control than the PWA group (*t* = −5.82, *p* < 0.001, *d* = −0.310, 95% CI [−0.002; −0.001], however, a model including PWA only still showed a significant increase in the proportion of passives produced across experimental trials (*t* = 4.48, *p* < 0.001, *d* = 0.353, 95% CI [0.001; 0.002].

#### 3.2.2. Cumulative eye fixation effects

**FIGURE 6:**
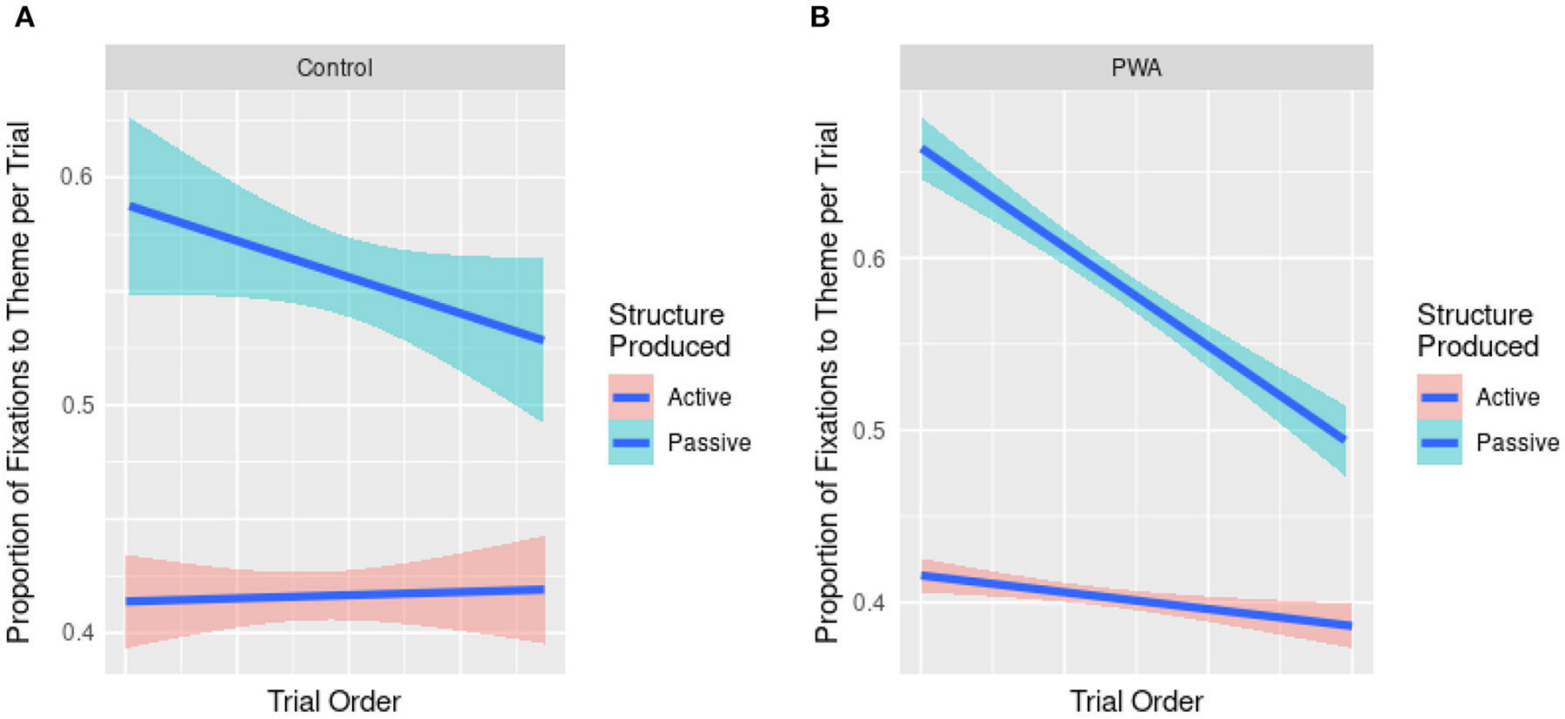
Plots of cumulative changes in fixations to the theme in the pre-onset region for controls **(A)** and PWA **(B)**.

Cumulative changes on fixation proportions to the theme are visualized in Figure 6 and model summaries are provided in Table 5. Structure produced was entered into these models as an additional predictor given that participants’ fixations to the agent or theme character are inherently tied to the active or passive word order they produced. Indeed, participants looked more to the theme in these early regions when producing passives compared to actives (*t* = 6.000, *p* < 0.001, *d* = 0.098, 95% CI [0.079; 0.156]). Crucially, PWA showed a stronger cumulative trend than controls as evidenced by a group * structure produced * trial order interaction (*t* = −2.388, *p* < 0.05, *d* = −0.039, 95% CI [−0.000; −0.000]. Indeed, after building separate models for PWA and control data, we found a significant Trial Order by Structure interaction in PWA (*t* = 2.086, *p* < 0.05, *d* = 0.039), but not in controls (*t* = −1.183, *p* > 0.05, *d* = −0.034). In the model including both groups’ data, pairwise *post-hoc* comparisons confirmed the greater cumulative facilitation when producing passives compared to actives (*z* = −15.21, *p* < 0.001). This was further shown in both groups separately, but with larger effects in the PWA than the control group (*z*_PWA_ = −23.542, *p* < 0.001; *z*_AM_ = −9.116, *p* < 0.001). These effects are further shown in Figure 6B.

We further ran analyses where aphasia test scores predicted cumulative priming effects. Predictor scores included the Aphasia Quotient of the Western Aphasia Battery (WAB AQ), and scores on the Verb Naming Test (VNT), Sentence Comprehension Test–Canonical (SCT-C), and Sentence Comprehension Test–Non-Canonical (SCT-NC). All these predictors were centered before being added to any model. Test scores were not predictive of cumulative priming effects in any behavioral model (all *p*s > 0.05). Similarly, on eye-tracking measures, none of the test scores predicted cumulative priming measures (all *p*s > 0.05). Our Supplementary material found at https://osf.io/7apc3/ contain the full code and results for these models.

## 4. Discussion

This study reports an eye-tracking-while-speaking structural priming investigation of Persons with Aphasia (PWA) and age-matched controls. Participants matched auditory primes consisting either of passive or active sentences to on-screen pictures, following which they described a transitive event. Specifically, we examined whether comprehension primes create immediate and lasting changes in participants’ behavioral sentence production and real-time (eye fixation) sentence planning processes. For immediate priming, we investigated whether PWA and controls showed a greater likelihood of producing passives and looking at the theme actor after comprehending a passive compared to an active prime. In addition, we examined if verb overlap between prime and target enhanced priming effects. For long-term effects, we investigated whether PWA and controls showed cumulative changes in their passive production and associated eye movements over the experimental session.

Overall, both controls and PWA were successfully primed in this experiment with cumulative priming effects being more consistent and robust in PWA than immediate priming effects. While immediate priming effects were significant in controls, PWA showed only a numerically greater tendency to produce passives immediately after passive vs. active primes. On eye fixation data, both PWA and controls spent on average a greater time fixating the theme character during target production in the passive vs. active prime condition, with this effect reduced for PWA. However, cumulative increases in the likelihood of producing passive compared to active structures were found in both groups. Both PWA and controls produced passives more frequently as the experiment progressed, and showed gradual changes in their eye fixations associated with the increased passive productions (see below for detailed discussion of cumulative priming effects). Despite the weak immediate priming effects found in PWA, these findings together suggest that structural priming remains intact in aphasia, in line with previous studies (Hartsuiker and Kolk, 1998; Cho-Reyes et al., 2016; Lee et al., 2019a; Man et al., 2019). Further, our finding of cross-modality priming, from comprehension to production, aligns with the findings of Keen and Lee (2022), who demonstrated structural priming from production to comprehension. These findings suggest that syntactic representations are shared between the comprehension and production modalities, and that structural priming in one modality facilitates access of these representations in the other modality in aphasia (Man et al., 2019; Keen and Lee, 2022).

While abstract structural priming was shown in both groups, the effects of verb overlap between prime and target were group–dependent. Significant lexical boost effects were found in the control group in both off-line production and eye fixation data. However, the lexical boost effect was not significant in PWA, suggesting that PWAs’ access to structural representations during sentence production may not be facilitated through lexical overlap. Our results concur with those of Lee et al. (2019a) and Man et al. (2019), who did not find lexical boost effects in PWA despite intact abstract priming. However, Yan et al. (2018) reported a significant lexical boost in PWA. The incongruency between our findings and Yan et al.’s (2018) may be explained in part by differences in priming modality. While our participants completed a comprehension task for prime sentences, the participants in the Yan et al. study were asked to verbally repeat prime sentences and to verify their own repetitions are correct against written prime sentences. It is possible that Yan et al.’s (2018) priming task allowed PWA to process lexical information with greater intensity, which may have affected the strength of the lexical boost (see further Lee et al., 2019a). Alternatively, the lexical boost appears more transient than abstract (different-verb) priming effects and may dissipate more quickly when intervening fillers or memory demands are at play (e.g., Hartsuiker et al., 2008; though cf. Van Boxtel and Lawyer, in preparation^1^). Thus, lexical information might have faded too quickly in our PWA, failing to generate additive priming during sentence production. Future studies investigating the lexical boost in PWA should therefore carefully manipulate experimental variables and cognitive-linguistic capacities of their participants. Generalizing our findings, abstract structural priming and the lexical boost may also be subserved by distinct cognitive mechanisms, which may be selectively impaired in PWA (see Lee et al., 2019a; Man et al., 2019, for additional evidence). This is a central tenet of dual-mechanism accounts of priming (Reitter et al., 2011; Chang et al., 2012; Traxler et al., 2014), which consider abstract priming the result of implicit learning, but in which the lexical boost is subserved by a transient activation-based or explicit memory-based mechanism. Tentatively, the current study lends some credence to these accounts.

Most importantly, however, both groups showed very clear cumulative priming effects on both behavioral and eye-tracking responses, making this study the first to report cumulative structural priming effects in PWA. As the experimental session progressed, both PWA and controls were more likely to describe transitive pictures using a passive structure. These significant cumulative increases in passive production indicate that through repeated processing of passive sentences both PWA and controls made an adaption in their grammatical encoding processes, resulting in increased use of passive structures over time. Further, these findings suggest that cumulative learning of syntactic structures remains preserved in aphasia.

In their pre-speech eye fixation data, PWA and controls spent increasingly less time fixating the theme during passive productions over the course of trials. Interestingly, this cumulatively reduced fixation time to the theme was found in tandem with both groups’ increased proportions of passive productions, but not when they produced active sentences. Because the reduced proportion of looking time to the theme actor also means increased looking time to the agent actor in the scene, this cumulative effect suggests that our participants were more likely to encode both theme and agent before speech onset, as they were producing passives more frequently and successfully. Such advanced encoding of both elements in the scene might facilitate efficient and accurate decision of the grammatical subject, easing subsequent sentence formulation processes (Bock and Griffin, 2000; Lee and Thompson, 2011; Van de Velde and Meyer, 2014; Lee et al., 2015; Mack et al., 2017; Lee, 2020). Thus, the current study shows that both off-line and real-time grammatical encoding of passives could be trained through structural priming.

Our exploratory considering individual patients’ baseline language testing scores revealed no significant interactions with cumulative priming effects. PWA exhibited priming regardless of the degrees of their syntactic deficits or aphasia severity. This is not entirely surprising given that many clinical tests of aphasia include explicit tasks. Further intact structural priming in spite of variability in aphasia testing scores suggests the ability to implicitly learn syntactic structures can remain intact in PWA, independent of impaired performance on clinical tests of aphasia.

The *greater* cumulative structural priming effects on eye fixation data found in PWA compared to controls deserve further attention. As Figure 6 shows, PWA exhibited a greater cumulative change in pre-speech fixations compared to controls. This may indicate that because the grammatical encoding system of PWA is weaker, they showed greater cumulative adaptation (or learning) during our structural priming task. Previous studies reported similar findings. Hartsuiker and Kolk (1998), for example, found that only PWA, but not controls, showed significant priming effects in their implicit structural priming conditions, although both groups showed significant priming effects when they were explicitly told to use the primed sentence structure in their target descriptions. The authors attributed this group difference to syntactic deficits in PWA, thus, allowing them to have more “room for improvement” than controls. Alternatively, controls, having greater pre-activation abilities of both active and passive structures at baseline than PWA, resulted in non-significant implicit priming effects. Similarly, Cho-Reyes et al. (2016) found that aphasic speakers with more severe language deficits showed larger priming effects in dative sentences, reflective of the error-based implicit learning mechanism underlying structural priming. Greater error, that is, a greater mismatch between a speaker’s predictions of an upcoming structure and the actual structure, leads to greater priming (Chang et al., 2012; Fine et al., 2013; Jaeger and Snider, 2013). Finally, the concept of “hyperpriming” has received attention in research on semantic priming in memory-impaired patients such as those with Alzheimer’s Disease (see van Boxtel and Lawyer, 2021, for a review). Put briefly, as a consequence of deteriorated connections between nodes in semantic memory, priming-induced activation might be allowed to spread much more rapidly and widely than in healthy individuals, resulting in stronger priming effects in patients.

Taken together, findings of intact structural priming in PWA are in line with other recent reports of successful priming in patient groups (Verreyt et al., 2013; Cho-Reyes et al., 2016; Yan et al., 2018; Lee et al., 2019a; Man et al., 2019, e.g.), suggesting the mechanisms underlying structural priming are intact in aphasia. Cumulative effects were especially telling in our PWA group, and indicate that gradual, implicit changes in the production systems of PWA remain possible even when immediate priming measures might seem small (Keen and Lee, 2022). Our study therefore supports models of structural priming rooted in implicit learning (e.g., Bock and Griffin, 2000; Chang et al., 2006, 2012; Traxler et al., 2014; Heyselaar et al., 2021), although given the absence of lexical boost effects in the PWA group, we are cautious about strongly supporting any one priming model. Crucially, however, our findings evidence that structural priming may induce lasting adaptation to linguistic encoding strategies (Pickering and Ferreira, 2008; Heyselaar and Segaert, 2022).

The current findings have important implications for developing structural priming as an intervention strategy, given ubiquitous difficulty with complex sentences such as passives in PWA (e.g., Bastiaanse and van Zonneveld, 2006; Cho and Thompson, 2010; Meyer et al., 2012). This study included only a single session for each participant, but even across one session we found that PWA showed cumulative changes in sentence production strategies and real-time grammatical encoding processes. In addition, because a structural priming paradigm such as the one used in this study does not require complex instructions or manipulation of materials, individuals who show deficits on explicit language tasks may still benefit from implicit priming. Adapting structural priming to a multi-session treatment could therefore be a cost-effective intervention for robust long-term and generalized treatment gains in PWA. Indeed, this notion has recently been explored in single-subject treatment (Benetello et al., 2012; Kalinyar-Fliszar et al., 2013; Lee and Man, 2017) and group studies (Lee et al., 2023), with promising early results.

Future investigations of priming effects in PWA should also include measures which explore generalization of priming effects to spontaneous speech. While the current study makes an important contribution to research on sentence production in aphasia through the comprehension-to-production priming paradigm we employed, we did not measure changes in the participants’ spontaneous speech. It therefore remains unclear whether priming effects generalize to spontaneous speech in PWA what modality priming should take to induce such changes. Finally, from our exploratory analyses, we did not discover meaningful associations between aphasia profiles and priming-induced changes on sentence production. More systematic investigations of person-specific characteristics and structural priming are necessary to understand the cognitive mechanisms underlying successful priming in aphasia, which will in turn inform which patients would benefit from structural priming interventions.

In conclusion, the current study investigated structural priming and lexical boost effects in PWA and healthy Controls, aiming to elucidate whether production of passive sentence structures was facilitated by preceding comprehension of passive primes, and whether concurrent eye movements were similarly affected. We found robust cumulative evidence for structural priming in both PWA and Controls on both behavioral and eye tracking data, suggesting priming can be an effective method for inducing facilitatory changes in the grammatical encoding systems of PWA. This study found no evidence for the lexical boost in PWA, indicating differences between the mechanisms underlying abstract structural priming and the lexical boost. All in all, we make the case for the further investigation of structural priming as a potential cost-effective treatment component for sentence production in aphasia.

## Figures and Tables

**TABLE 1:** Language testing scores from participants with aphasia.

PWA	Western Aphasia Battery Revised (WAB–R)					Northern Assessment of Verbs and Sentences (NAVS)						
	Fluency	AC	Rep	Naming	AQ	VNT	VCT	ASPT	SPPT-C	SPPT-NC	SCT-C	SCT-NC
1	5	8.7	4.4	7.7	69.6	72.7	100	100	13.3	0	93.3	26.7
2	6	9.1	9	9.2	84.6	100	100	97	93	80	100	93
3	5	10	9.2	8.3	83	97	100	100	100	93	100	100
4	6	8.8	7.4	7.3	77	50	100	93.9	80	6.7	80	60
5	9	9.9	8.6	9.1	93.1	95.5	100	100	100	100	100	100
6	9	10	9.4	9.7	96.2	100	100	100	100	100	100	100
7	8	9.3	9.5	6.7	85	95	100	100	93.3	80	80	73
8	4	8.6	10	6	73	13.6	90.9	56.3	53.3	60	86.7	80
9	8	9.6	8.4	8.9	87.7	83	100	93.8	100	67.7	93.3	86.7
10	9	9.2	9	10	94.4	100	100	100	100	93.3	100	100
11	9	10	9.4	9.7	96.2	100	100	100	100	100	100	80
12	6	8.5	6.7	8.4	75.2	81.8	95.5	68.8	33.3	20	60	53.3
13	8	9.6	10	6.8	86.7	81.8	100	96.9	100	100	93.3	93.3
14	5	10	5.1	7.3	68.8	86.4	95.5	93.8	100	60	100	100
15	6	9.1	4.6	8.5	72.3	90.9	100	90.6	60	0	93.3	80
16	5	9.5	9.1	9.2	81.6	60	100	90	100	86.7	100	53.3
Mean	6.8	9.4	8.1	8.3	82.8	81.7	98.9	92.5	82.9	65.5	92.5	80.0
SD	1.8	0.5	1.9	1.1	9.4	23.4	2.6	12.5	27.8	37.7	11.1	21.7

AC, Auditory Comprehension; Rep, Repetition; AQ, Aphasia Quotient; VNT, Verb Naming Test; VCT, Verb Comprehension Test; ASPT, Argument Structure Production Test; SPPT-C, Sentence Priming Production Test–Canonical; SPPT-NC, Sentence Priming Production Test–Non-canonical; SCT-C, Sentence Comprehension Test–Canonical; SCT-NC, Sentence Comprehension Test–Non-canonical.

**TABLE 2:** Generalized linear mixed model summary predicting active or passive responses on target productions.

Parameter	Est.	SE	*z*	*p*	*Partial R* ^2^	*Upper CL*	*Lower CL*
Intercept	−3.539	0.455					
Group	1.489	0.572	2.603	**0.009**	0.012	0.025	0.003
Prime	2.192	0.406	5.405	**<0.001**	0.026	0.044	0.012
Verb	−0.678	0.579	−1.172	0.241	0.003	0.011	0.000
Group * prime	−1.850	0.509	−3.634	**<0.001**	0.009	0.021	0.002
Group * verb	0.770	0.658	1.171	0.241	0.002	0.008	0.000
Prime * verb	2.153	0.634	3.395	**0.001**	0.013	0.027	0.004
Group * prime * Verb	−1.992	0.760	−2.620	**0.009**	0.005	0.015	0.000

Model formula: Structure produced ~ group*prime*verb + (1 ∣ Participant); random effect for participant (*σ*^2^ = 1.04, SD = 1.02). Est, estimate; SE, standard error; Upper/Lower CL, 95% confidence interval limits. *R*^2^_Marginal_ = 0.275, *R*^2^_Conditional_ = 0.456. Values meeting the significance threshold of *p* < 0.05 are represented in bold.

**TABLE 3:** Linear mixed model summary predicting the proportion of time spent fixating the theme in the pre-onset region during production of passives only.

Parameter	Est.	SE	*t*	*p*	*d*	Upper CL	Lower CL
Intercept	0.497	0.168					
Prime	0.036	0.033	1.079	0.281	0.043	0.101	−0.030
Verb	0.127	0.049	2.566	**0.010**	−0.031	0.223	0.030
Group	0.047	0.049	0.963	0.339	−0.079	0.142	−0.048
Prime * verb	−0.015	0.052	−2.032	**0.043**	0.073	−0.003	−0.205
Prime * group	−0.028	0.038	−0.075	0.451	0.032	0.046	−0.101
Verb * group	−0.161	0.052	−3.111	**0.001**	0.038	−0.059	−0.262
Prime * verb * group	1.695	0.056	3.009	**0.003**	−0.040	0.279	0.059
Years in education	0.0001	0.009	0.012	0.990	−0.225	0.017	−0.048

Model formula: Theme proportion ~ prime*verb*group + years in education + (1 ∣ trial) + (1 ∣ participant) + (1 ∣ agent position); random effects for participant (*σ*^2^ = 0.006, SD = 0.077); trial (*σ*^2^ = 0.006, SD = 0.079); and agent position (*σ*^2^ = 0.011, SD = 0.104). Est, estimate; SE, standard error; Upper/lower CL, 95% confidence interval limits. *R*^2^_Marginal_ = 0.010, *R*^2^_Conditional_ = 0.373. Values meeting the significance threshold of *p* < 0.05 are represented in bold.

**TABLE 4:** Linear mixed model summary predicting the cumulative proportion of passives produced across experimental trials.

Parameter	Est.	SE	*t*	*p*	*d*	Upper CL	Lower CL
Intercept	−0.011	0.096					
Trial order	0.003	0.0002	15.758	**<0.001**	0.842	0.003	0.003
Group	0.059	0.040	1.467	0.150	0.461	0.138	−0.020
Trial order * group	−0.002	0.003	−5.82	**<0.001**	−0.310	−0.001	−0.002
Years in education	0.006	0.005	1.044	0.297	0.113	0.016	−0.005

Model formula: Cumulative passive proportion ~ trial order*group + years in education + (1 ∣ participant); random effect for participant (*σ*^2^ = 0.011, SD = 0.103). Est, estimate; SE, standard error; Upper/Lower CL, 95% confidence interval limits. *R*^2^_Marginal_ = 0.068, *R*^2^_Conditional_ = 0.695. Values meeting the significance threshold of *p* < 0.05 are represented in bold.

**TABLE 5:** Linear mixed model summary predicting the cumulative proportion of fixation time to the theme across experimental trials.

Parameter	Est.	SE	*t*	*p*	*d*	Upper CL	Lower CL
Intercept	4.213	0.051					
Trial order	−0.000	0.000	−0.790	0.430	−0.013	0.000	−0.000
Group	−0.026	0.018	−1.407	0.167	−0.414	0.010	−0.061
Structure produced	0.117	0.020	6.000	**<0.001**	0.098	0.156	0.079
Trial order * group	0.000	0.000	1.077	0.291	0.017	0.000	−0.000
Trial order * structure produced	0.091	0.000	0.564	0.573	0.009	0.001	−0.001
Group * structure produced	0.091	0.022	4.157	**<0.001**	0.068	0.136	0.048
Trial order * group * structure produced	−0.001	0.000	−2.388	**0.017**	−0.039	−0.000	−0.000
Years in education	−0.003	0.004	−0.830	0.413	−0.039	0.004	−0.010

Model formula: Theme proportion ~ trial order * group * structure produced + years in education + (1 ∣ participant) + (1 ∣ agent position); random effects for participant (*σ*^2^ = 0.001, SD = 0.34) and agent position (*σ*^2^ = 0.005, SD = 0.070). Est, estimate; SE, standard error; Upper/Lower CL, 95% confidence interval limits. *R*^2^_Marginal_ = 0.048, *R*^2^_Conditional_ = 0.140. Values meeting the significance threshold of *p* < 0.05 are represented in bold.

## Data Availability

The datasets presented in this study can be found in online repositories. The names of the repository/repositories and accession number(s) can be found below: https://osf.io/7apc3/.

## Footnotes

1 Van Boxtel, W. S., and Lawyer, L. A. (in preparation). *Persistence of the Lexical Boost in Syntactic Comprehension Priming*. Available online at: https://osf.io/8eb3a/
